# Supplementary material for: Community Health Survey of Residents Living Near a Solid Waste Open Dumpsite in Sabak, Kelantan, Malaysia
Source: Int J Environ Res Public Health. 2020 Jan 2;17(1):311. doi: 10.3390/ijerph17010311 (PMC6981880; doi:10.3390/ijerph17010311)
Supplement: Supplementary file 1 [file ijerph-17-00311-s001.pdf]

QUESTIONNAIRES ON HEALTH STATUS OF RESIDENTS LIVING NEAR SABAK DUMPSITE

A. SOCIO-DEMOGRAPHY

Addresss:____

Tel no:___________________ Birthdate:______________ Age:____________

Sex: Male ☐ Female ☐

Race: Malay ☐ Chinese ☐ Indian ☐ Siamese ☐ Others ☐

Occupation: Housewife ☐ unemployed ☐ private sector ☐ government sector ☐ self-employed ☐

State your occupation :_______________________

Do you work in factory?, *state type of factory*__________________________

Total monthly household income (RM): _______________________

Education level: none ☐ SRP / PMR ☐ SPM ☐ STPM/Diploma ☐ degree/post-graduate ☐

Date begin living in this village (minimum one year):__________________

Source of drinking water: Tube well ☐ Dug well ☐ Kelantan Water ☐ Others ☐, state_____________

Do you smoke? Yes ☐ No ☐ Ex-smoker ☐

If yes, how many stick per day ___________ *If previous smoker, how long you have quitted*? ___________

Any family member who smoke? Yes ☐ No ☐ if yes, *how many?*_____

Do you regularly collect any waste from the landfill? Yes ☐ No ☐

Do you grow your own vegetables for own consumption? Yes ☐ No ☐

Do you breed chickens for own consumption? Yes ☐ No ☐

B. SYMPTOMS

Have you / your family experienced the following problems in the last 3 months and how often?

		**Yes**	**If yes, who? How often?**	**No**
1.	Itching or irritation in the eyes	☐	____________________	☐
2.	Skin rashes	☐	____________________	☐
3.	Itching or irritation of nose	☐	____________________	☐
4.	Headache	☐	____________________	☐

5. Excessive tiredness of doing daily chores ☐ ____________________ ☐

6. Excessive day time sleepiness ☐ ____________________ ☐

7. Sore throat ☐ ____________________ ☐

8. Diarrhea ☐ ____________________ ☐

9. Stomachache ☐ ____________________ ☐

C. DIAGNOSED DISEASES/ILLNESS

Have you / your family member been or are currently suffering from the following illnesses?

**Disease certified by a doktor**	**Yes**	**No**	**Date diagnosed**	**Date admission in hospital & reason?**
Tuberculosis (TB)				
Asthma				
Pneumonia				
Typhoid fever				
Cholera				
Dengue fever				
Hepatitis A				
Food poisoning				
Diabetes mellitus				
Hypertension				
Cancer, *state site*				
Ischemic eart disease				
Epilepsy				
Enuresis (children)				
Learning problem (children)				
Hyperactive children				

D. CHILD AND MATERNAL HEALTH

No of children: ________ No of living children_______

Miscarriage(1-9-1996 till now): Yes ☐ No ☐ If yes, state when & number of miscarriage ________

No of child death (1-9-1996 till now) ________

Please provide details of children born 1-9-1996 to the present (or have been living in the area for at least a year). See the antenatal card or hospital card.

BIRTHDATE	SEX M/F	BIRTH WEIGTH (kg)	GESTATION (wk)	MOTHER AGE	COMPLICATION DURING PREGNANCY (Yes/No) State details	CONGENITAL ANOMALIES (Yes/No) State details	DEATH <5y (Yes/No) State detail

THANK YOU FOR YOUR COOPERATION
